# Supplementary material for: Comprehensive Annotation and Functional Exploration of MicroRNAs in Lettuce
Source: Front Plant Sci. 2021 Dec 24;12:781836. doi: 10.3389/fpls.2021.781836 (PMC8739914; doi:10.3389/fpls.2021.781836)
Supplement: Supplementary file 2 [file Data_Sheet_8.ZIP › GSM2230747.plot/Lsa-miRN565_Lsat_1_v5_gn_2_41661.1_2064_TPlot.pdf]

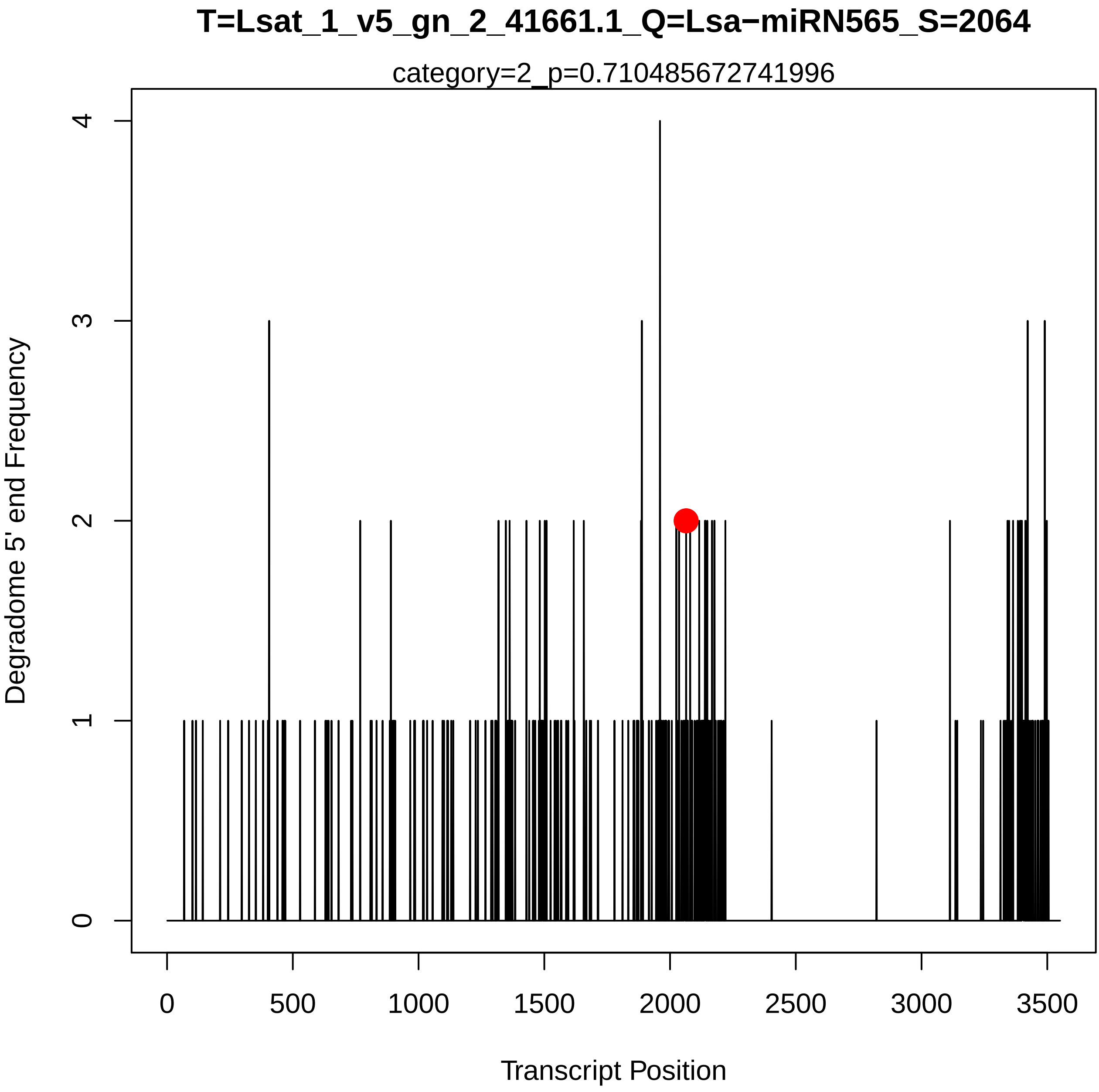
T=Lsat_1_v5_gn_2_41661.1_Q=Lsa−miRN565_S=2064
category=2_p=0.710485672741996
Degradome 5' end Frequency
0
1
2
3
4
0
500
1000
1500
2000
2500
3000
3500
Transcript Position
